# Temporal filtering of luminance and chromaticity in macaque visual cortex

**DOI:** 10.1016/j.isci.2021.102536

**Published:** 2021-05-18

**Authors:** Gregory D. Horwitz

**Affiliations:** 1 Department of Physiology and Biophysics, Washington National Primate Research Center, University of Washington, 1959 N.E. Pacific Street, HSB I-714, Box 357290, Seattle, WA 98195, USA

**Keywords:** Biological sciences, Neuroscience, Sensory neuroscience

## Abstract

Contrast sensitivity peaks near 10 Hz for luminance modulations and at lower frequencies for modulations between equiluminant lights. This difference is rooted in retinal filtering, but additional filtering occurs in the cerebral cortex. To measure the cortical contributions to luminance and chromatic temporal contrast sensitivity, signals in the lateral geniculate nucleus (LGN) were compared to the behavioral contrast sensitivity of macaque monkeys. Long wavelength-sensitive (L) and medium wavelength-sensitive (M) cones were modulated in phase to produce a luminance modulation (L + M) or in counterphase to produce a chromatic modulation (L − M). The sensitivity of LGN neurons was well matched to behavioral sensitivity at low temporal frequencies but was approximately 7 times greater at high temporal frequencies. Similar results were obtained for L + M and L − M modulations. These results show that differences in the shapes of the luminance and chromatic temporal contrast sensitivity functions are due almost entirely to pre-cortical mechanisms.

## Highlights

• Near-threshold, periodic light modulations drive frequency-specific spiking in LGN
• High-frequency signals are filtered cortically, whether color or luminance
• The retina, not cortex, mediates temporal differences between color and luminance

## Graphical abstract

**Figure:**
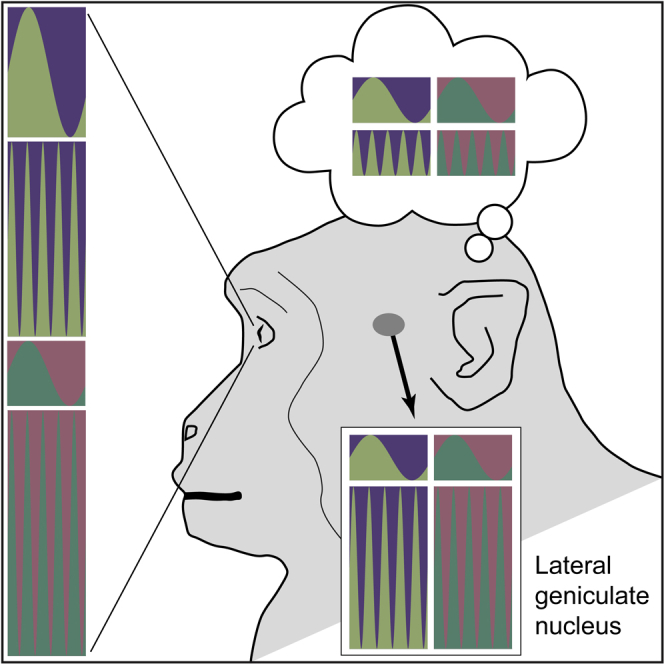

## Introduction

Signal processing in the visual system preserves some types of information while eliminating others. If perfect knowledge of neuronal activity at one stage of the visual system (e.g. visual cortex) allows for perfect reconstruction of activity at an earlier stage (e.g. the photoreceptors), then information is perfectly preserved between them. If, instead, multiple patterns of activity at an early stage produce indistinguishable patterns at a later stage, then information has been lost. The ability of an observer to detect a stimulus—to distinguish it from a blank—is a consequence of information that is retained throughout the entire visual system. A central goal of visual neuroscience is to understand which types of information are lost and at which stage.

A salient example of information loss in the visual system is the dependence of the visibility of a periodic stimulus on its temporal frequency. This relationship, the temporal contrast sensitivity function, plays important roles in industry (Legall, 1991) and medicine (Owsley, 2011; Tyler, 1981), but its biological basis is incompletely understood. This uncertainty is due in part to methodological differences between neurophysiological and behavioral studies. Temporal contrast sensitivity depends on many factors including observer species, background luminance, retinal eccentricity, stimulus size, and duration (Benardete and Kaplan 1997b, 1999a; Lee et al., 1990; Lindbloom-Brown et al., 2014; Merigan, 1980; Pokorny et al., 2001; Snowden and Hess, 1992; Snowden et al., 1995; Solomon et al., 2002; Swanson et al., 1987; Van der Horst 1969). In the current study, care was taken to match these factors between neurophysiological and behavioral measurements, providing a clearer picture of their relationship than has previously been available.

**Figure 1:**
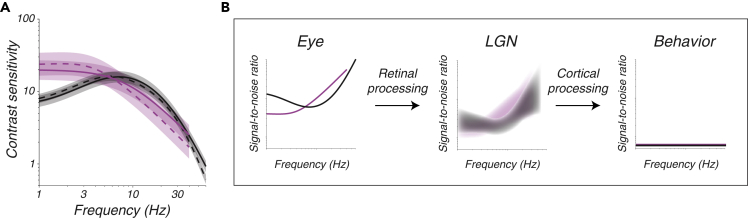
Temporal contrast sensitivty and experimental logic (A) Temporal contrast sensitivity functions from monkey 1 (dashed) and monkey 2 (solid) for L + M modulations (black) and L − M modulations (magenta). Curves represent the means across receptive field locations of recorded LGN neurons, and bands represent ±1 standard deviation. (B) Schematic of the experimental logic. A set of stimuli, which varied in relative L and M cone phase (L + M or L − M) and temporal frequency, was presented at the RF of each neuron studied. The contrast of each stimulus (left) was adjusted to equate the signal-to-noise ratio at the level of behavior (right). Predictions in the middle panel are fuzzy to depict uncertainty in the signal-to-noise ratio of responses in the LGN.

Chromatic modulations are easier to see than luminance modulations at low temporal frequencies, but at higher frequencies, the reverse is true (De Lange Dzn, 1958; Kelly and van Norren, 1977) (Figure 1A). The photoreceptors cannot be responsible for this difference because the same photoreceptor types that mediate luminance detection also mediate chromatic detection. Luminance stimuli modulate the long- (L) and medium wavelength-sensitive (M) cones in phase (L + M), whereas most chromatic stimuli modulate them in counterphase (L − M). Differences in the temporal filtering of these two stimulus classes must therefore be due to stages of the visual system where signals from the L and M cones are already mixed.

The purpose of this study was to quantify cortical and pre-cortical contributions to luminance and chromatic temporal contrast sensitivity. Cortical contributions were computed by comparing the behavioral sensitivity of a macaque monkey to that of a computational observer of spikes in the lateral geniculate nucleus (LGN) (Figure 1B). Pre-cortical contributions were computed by comparing two computational observers: one of LGN spikes and one of simulated currents across the outer segments of modeled cone photoreceptors.

The main result of these comparisons was that information loss in the cortex was similar for L + M and L − M modulations, whereas information loss between the cones and LGN differed profoundly for L + M and L − M modulations. Differences in luminance and chromatic behavioral temporal contrast sensitivity are therefore due to processes occurring upstream of the LGN with minimal cortical involvement.

## Results

Two monkeys (*M. mulatta*) performed a 2-alternative, forced-choice contrast detection task that required them to report on which side of a computer screen a drifting Gabor stimulus appeared. Detection thresholds were measured as a function of stimulus location, temporal frequency, and the amplitude of L and M cone modulations. A model was developed that predicted detection thresholds as a function of all of these parameters jointly (Gelfand and Horwitz, 2018 and Figure 1A). Visual stimuli were constructed on the basis of this model and used to measure the signal-to-noise ratio (SNR) of LGN neuronal responses (Figure 1B). All stimuli were at the monkeys' behavioral detection threshold, or equivalently, matched for SNR at the output of the visual system.

### LGN single-unit responses

The spatial and spectral sensitivity of each recorded LGN neuron were characterized with a white noise stimulus (Horwitz, 2020). Spike-triggered averaging was used to locate the receptive field (RF) center and to identify the physiological type of each neuron. Fifteen neurons were classified as magnocellular (8 from monkey 1 and 7 from monkey 2) and 38 as parvocellular (19 from each monkey). Each recorded neuron was then stimulated with Gabor patterns centered on its RF that varied across trials in temporal frequency and L and M cone modulation phase (in-phase, L + M, or counterphase, L − M). The L and M cone contrasts were always equal, and their maximum was set by the limits of the display (0.19 for the L − M stimulus and 0.86 for the L + M stimulus). A blank stimulus was included to measure baseline firing statistics.

**Figure 2:**
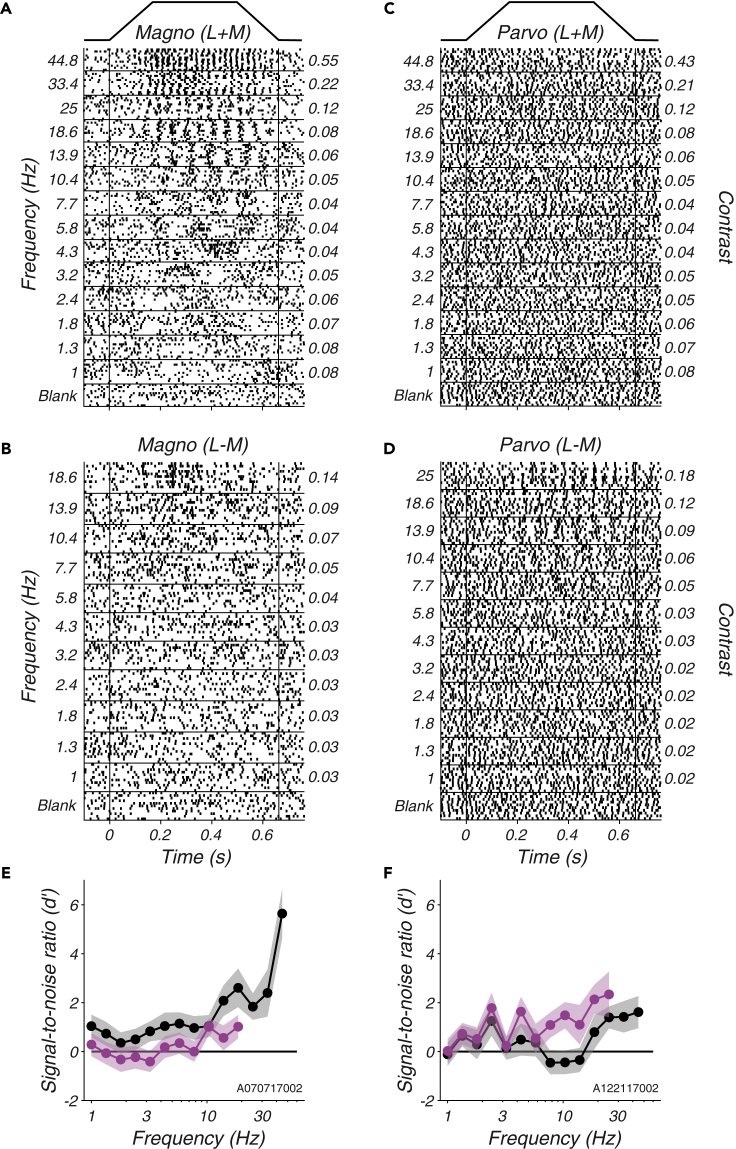
Responses of two LGN neurons to Gabor stimuli near behavioral detection threshold (A) Raster plot of magnocellular responses to L + M modulations. Trials have been sorted by temporal frequency (left ordinate) which covaries with cone contrast (right ordinate, identical for L and M cones) to maintain a constant level of stimulus detectability. The temporal envelope of the Gabor stimulus is shown above the rasters. (B) Identical to (A) but showing responses to L − M modulations. (C and D) Identical to (A) and (B) but for a parvocellular neuron. (E) Signal-to-noise ratio (d') calculated from responses in (A) (black) and from responses in (B) (magenta). Shaded bands represent ±1 standard error estimated by non-parametric bootstrap. (F) Identical to (E) but for the parvocellular neuron. See also Figures S1 and S2.

A representative magnocellular neuron responded to L + M modulations vigorously at high temporal frequencies and more weakly as temporal frequency was reduced (Figure 2A) (for similar data from a second magnocellular neuron, see Horwitz, 2020). This neuron also responded to L − M modulations but only at the highest frequencies tested and then only transiently (Figure 2B). An example parvocellular neuron responded more vigorously to L − M modulations than to L + M modulations (Figures 2C and 2D), although, as expected from their low contrast, none of the stimuli used in this study drove parvocellular neurons strongly (the example in Figures 2C and 2D is among the most responsive parvocellular neurons in the data set).

The SNR of each response was calculated by comparing it to baseline activity. This analysis assumes that the signal in the spike trains is at the fundamental temporal frequency of the stimulus (see supplemental information, transparent methods), but this assumption was not critical to the main results (see supplemental information, Figures S1 and S2). The example magnocellular neuron had greater SNR for L + M than for L − M modulations at all frequencies tested (Figure 2E). The example parvocellular neuron had greater SNR for L − M modulations than for L + M modulations above 6 Hz (Figure 2F).

**Figure 3:**
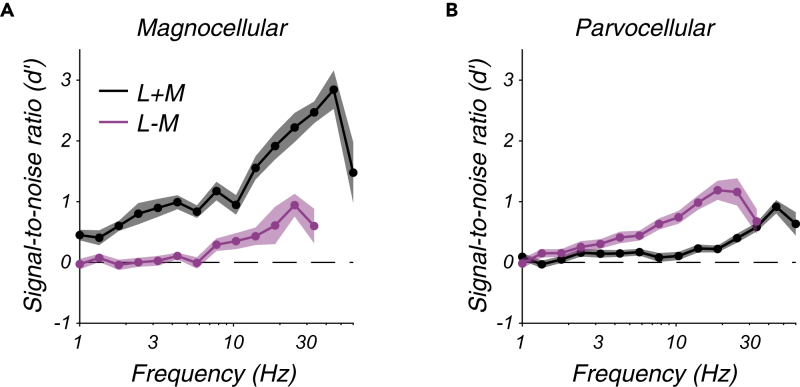
Population signal-to-noise analysis (A and B) Signal-to-noise ratio (d') averaged across magnocellular neurons (A) and parvocellular neurons (B). Points represent means across neurons, and shaded bands represent ±1 standard error of the mean.

The relationships among spiking responses, temporal frequency, L and M cone modulation, and cell type become clearer when data are averaged across neurons (Figure 3). As expected, magnocellular neurons were more sensitive to L + M modulations, and parvocellular neurons were more sensitive to L − M modulations (Wiesel and Hubel, 1966). The SNR of magnocellular and parvocellular responses increased smoothly from 1 to 20 Hz despite the fact that contrast changed with temporal frequency in different ways for L + M and L − M modulations over this range to render each stimulus near detection threshold.

The SNR of the average neuron (Figure 3) is lower than the SNR of neuronal populations. To estimate the SNR of a population of LGN neurons, the SNR of individual LGN neurons was inflated by an estimate of how many LGN neurons were modulated by the stimulus, as described in the next section.

### Population SNR analysis

Magnocellular neurons have greater contrast sensitivity than parvocellular neurons do at matched eccentricity, but they are less numerous, raising the possibility that, as populations, parvocellular neurons might have greater SNR (Croner and Kaplan, 1995). To estimate the SNR of neuronal populations, a model was constructed using parameters from the literature (Horwitz, 2020). The model provided a scale factor for each neuron that reflects how many times greater the SNR of a population of similarly sensitive neurons is. Scale factors were 2.1-fold greater (±0.4 SD) for parvocellular neurons than magnocellular neurons at matched eccentricity.

**Figure 4:**
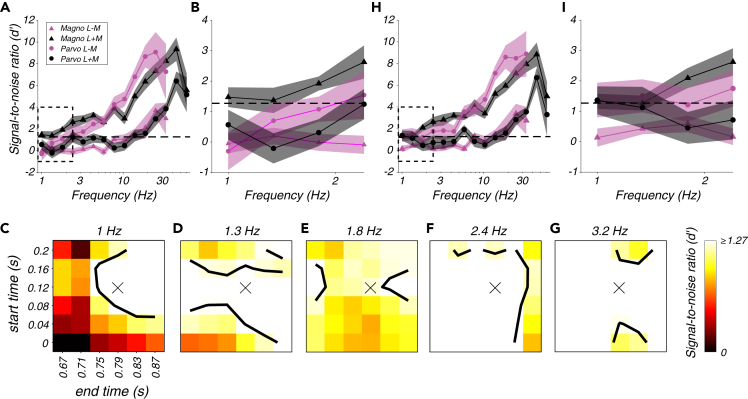
Population signal-to-noise analysis (A and B) (A) Population signal-to-noise ratio (d') as a function of temporal frequency for magnocellular neurons (triangles) and parvocellular neurons (circles) in response to L + M modulations (black) and L − M modulations (magenta). Bands represent ±1 standard error of the mean across neurons. Dashed line at 1.27 indicates the d' inferred from behavioral sensitivity. Dashed rectangle represents the region magnified in (B). (C) Population d' for parvocellular neurons in response to 1 Hz, L − M modulations as a function of the start time (ordinate), and end time (abcsissa) of the spike counting window. Contour is drawn at d' = 1.27. A spike counting window delayed by 120 ms from the stimulus presentation epoch (marked by an "X") produced a greater d' value than the window used in (A) and (B), which did not take response latency into account (lower left corner). (D–G) Identical to (C) but for 1.3, 1.8, 2.4, and 3.2 Hz modulations, respectively. (H and I) Identical to (A) and (B) but counting spikes from 120 ms after the stimulus appeared until 120 ms after the stimulus disappeared. See also Figure S3.

Parvocellular population SNR rose steeply with the temporal frequency of L − M modulations, and magnocellular population SNR rose similarly with the temporal frequency of L + M modulations (Figure 4A). Magnocellular and parvocellular populations were also weakly and similarly responsive to their non-preferred modulations, L − M and L + M, respectively. The similarity of these patterns is striking considering that these data were derived from recordings from two distinct populations of neurons responding to two sets of stimuli that varied in temporal frequency and L and M cone contrast in different ways.

To quantify how much temporal information was lost in the cortex, SNR in the LGN was compared to behavioral sensitivity. For this purpose, the monkeys' percent correct at threshold, 82%, was converted to an SNR of 1.27 (Green and Swets, 1966, see methods). At high frequencies, the SNR of magnocellular and parvocellular neurons exceeded this level by approximately a factor of 7 in response to L + M and L − M modulations, respectively. At lower frequencies, SNR in the LGN was lower. In fact, at the lowest frequencies tested, parvocellular population SNR fell below behavioral SNR for both L + M and L − M modulations (Figure 4B and methods). Parvocellular neurons are the sole conduit by which low temporal frequency L − M modulations are transmitted from the eye to the cortex, so parvocellular SNR was underestimated.

The analysis in Figures 4A and 4B was based on spikes recorded between stimulus onset and disappearance, including the slow (166 ms) contrast ramps at the beginning and end of each stimulus presentation. No adjustment was made for response latency, which biased SNR downward. To examine the effects of spike counting window on SNR, the start and stop times for spike inclusion were varied independently over a 200-ms range (Figures 4C–4G). This analysis showed that delaying the spike counting window by ∼120 ms relative to the stimulus presentation boosted parvocellular population SNR sufficiently to mediate behavior at even the lowest temporal frequencies tested. This delay presumably reflects the low contrast sensitivity of parvocellular neurons combined with the slow contrast increase at the beginning of each stimulus presentation.

Across cell types and stimulus conditions, delaying the spike counting window by 120 ms affected SNR only subtly (compare Figures 4A to 4H and 4B to 4I). Over a broader range of spike counting windows, none was found that rendered parvocellular populations significantly more sensitive to low-frequency L − M modulations than the monkey (Figure S3). Over the same range of windows, magnocellular and parvocellular population SNRs were similar for L + M and L − M modulations, respectively (Figure S3).

These analyses show that low-frequency information was preserved with near-perfect fidelity downstream of the LGN, and the amount of information lost downstream of the LGN was nearly independent of whether L and M cone modulations were in phase or in counterphase. The difference between the luminance and chromatic temporal contrast sensitivity functions is therefore due primarily to information loss upstream of the LGN, which is quantified next.

### SNR loss upstream of the LGN

**Figure 5:**
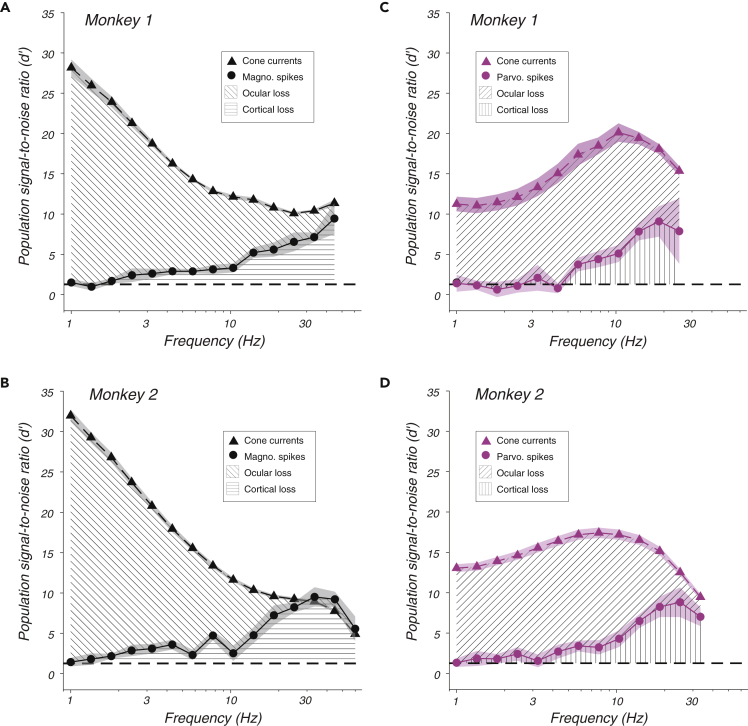
Signal loss from cones to LGN to behavior Population signal-to-noise ratio (d') for monkey 1 (A and C) and monkey 2 (B and D). Symbols represent means across neurons, and shaded bands represent ±1 standard error of the mean. Population d' was calculated from simulated cone currents (triangles) and recorded LGN spikes (circles) in response to L + M modulations (black) and L − M modulations (magenta). Diagonal cross-hatching shows the difference in d' between cone currents and LGN spikes. Horizontal and vertical cross-hatching shows the difference in d' between LGN spikes and behavior. See also Figure S4.

To measure how much information was lost between the cone photoreceptors and the LGN, cone photocurrent responses to the stimuli used in the LGN recordings were simulated using the model of Angueyra and Rieke, (2013). SNR loss between the cones and the LGN exceeded SNR loss in the cortex and was particularly severe at low temporal frequencies (Figure 5, diagonal cross hatches). Only 5% of the SNR available in cone outer segment currents in response to low-frequency L + M modulations reached the LGN (Figures 5A and 5B). In response to L − M modulations, information transmission efficiency was more than doubled (Figures 5C and 5D). Above 5 Hz, the situation reversed; SNR loss for L − M modulations exceeded SNR loss for L + M modulations. This analysis confirms differential retinal filtering of L + M and L − M modulations and shows that most of the information loss under the conditions tested occurred upstream of the LGN.

## Discussion

Much of the information in the light absorbed by photoreceptors fails to reach perception (Barlow, 1957; Geisler, 1989, 2011). Identifying where and how this information is lost is a key step toward understanding the biological basis of vision. The distinctive temporal properties of luminance and chromatic vision offer insight into this broader issue. The fact that information loss is temporal, not spatial, indicates a neural basis as opposed to an optical one. The fact that the same photoreceptor types mediate both aspects of vision indicates that the information loss is downstream of the photoreceptors. Previous studies have shown that low-frequency L + M modulations are selectively filtered in the retina and that high-frequency modulations are filtered in the cortex (Kaplan and Benardete, 2001; Kaplan et al., 1990). Contributions of the current study are the quantitative comparison of information loss upstream and downstream of the LGN and the demonstration that cortical filtering of L + M and L − M modulations is similar across temporal frequencies.

### Mechanisms of SNR loss in the retina and LGN

**Figure 6:**
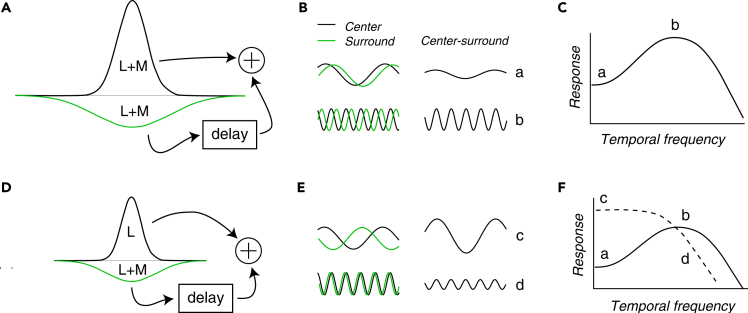
Temporal filtering by center-surround receptive field antagonism (A) Schematic receptive field profile of an ON-center cell. Center (narrow upright Gaussian) and surround (broad upside down Gaussian) are sensitive to a sum of L and M cone modulations. (B) Modulations of the center (black) and surround (green) in response to L + M modulations (left) are subtracted (right) to represent the net response to a stimulus that modulates both center and surround together. (C) Temporal frequency tuning of the neuron in (A). (D–F) Similar to (A–C) but for an L-ON cell. Traces in (E) represent responses to L − M modulations. Dashed curve in (F) represents temporal frequency tuning for L − M modulations. a, b, c, and d in (B) and (E) denote stimuli that correspond to points on the temporal frequency tuning curves in (C) and (F).

The stimuli used in this study were approximately uniform within the RF of each LGN neuron studied. Consequently, center-surround antagonism reduced SNR in response to L + M modulations at low temporal frequencies (Figures 6A and 6B, top). At higher temporal frequencies, the delay of the surround became an appreciable fraction of the stimulus period, causing excitation from the center to move closer in time to the release of surround inhibition (Enroth-Cugell et al., 1983; Robson, 1966) (Figure 6B, bottom). This change in the relative timing of excitation and inhibition largely explains the weaker response of LGN neurons to low frequency L + M modulations than to higher frequency (5–10 Hz) L + M modulations (Benardete and Kaplan, 1997a, 1999a) (Figure 6C).

Most parvocellular neurons with parafoveal RFs receive input from a single cone type to the center of their RFs and a mixture of L- and M-cones to the surround. For these neurons, L − M modulations invert the influence of the surround relative to the center. A parvocellular L-ON neuron, for example, is excited by an increase in L cone contrast at the center and is disinhibited by a decrease in M cone contrast in the surround (Figure 6D). When close together in time, these influences combine to drive a strong response (Figure 6E, top). When the temporal frequency of the modulation is sufficiently high that excitation from the center coincides with inhibition from the surround, the response is reduced (Figure 6E, bottom & 6F). These center-surround interactions explain the low-pass temporal frequency tuning of parvocellular neurons to L − M modulations (Benardete and Kaplan, 1999b; Lankheet et al., 1998).

The high-frequency roll-off of magnocellular and parvocellular responses is due largely to phototransduction, the dynamics of which depend on mean light intensity. Across a broad range of light levels, increasing the mean intensity of a modulated light increases the speed of cone responses (Baudin et al., 2019) and retinal ganglion cell (RGC) responses (Purpura et al., 1990) and shifts the peak of the psychophysical temporal contrast sensitivity function to higher frequencies (De Lange Dzn, 1958). The ability to predict the shape of the high-frequency limb of the temporal contrast sensitivity function on the basis of the cone temporal impulse response across light levels suggests that that cortical filtering is independent of light level (Lee et al., 1990; Rider et al., 2019; Stockman et al., 2006).

Temporal filtering at the retinogeniculate synapse appears to be modest under most conditions (Alitto and Usrey, 2008; Benardete and Kaplan, 1997a, 1999b; Kaplan et al., 1987; Kaplan and Shapley, 1986). Many of the stimuli used in the current study had low contrast, making retinogeniculate transmission particularly efficient (Kaplan et al., 1987). High-frequency stimuli had higher contrasts, but the similarity in SNR of cone currents and LGN spiking responses at these frequencies suggests that information loss at the retinogeniculate synapse was minimal.

### Mechanisms of SNR loss in the cortex

The SNR gap between the LGN and behavior is due, at least in part, to processes occurring in area V1 (Hawken et al., 1996). One mechanism that may contribute to high-frequency filtering in V1 is push-pull excitation-inhibition (Tolhurst and Dean, 1990). Simple cells in V1 receive spatially coincident excitation and inhibition that prevent high-contrast, non-preferred stimuli from driving a response (Troyer et al., 1998) and reduce sensitivity to high temporal frequency modulations (Krukowski and Miller, 2001; Krukowski et al., 2001). An intuition for the latter effect is that excitation and inhibition cancel when triggered simultaneously. The dominant inhibition required by the push-pull model ensures that cancellation is complete, and the slow kinetics of N-methyl-D-aspartate-sensitive channels in V1 neurons broaden the window of effective simultaneity (Eickhoff et al., 2007; Lester et al., 1990).

Most of the data supporting the push-pull model are from cat, but the same principles are likely at work in primates as well (Conway and Livingstone, 2006; Kremkow and Alonso, 2018). Monkeys have luminance-tuned simple cells, like cats do, but unlike cats, monkeys have a large population of cone-opponent V1 neurons. Some of these cone-opponent neurons combine visual signals antagonistically and roughly linearly across their RFs, consistent with the push-pull model (Conway and Livingstone, 2006; De and Horwitz, 2021). One possibility that is consistent with the results of this study is that push-pull excitation-inhibition reduces the SNR of high-frequency cone-opponent and non-opponent modulations similarly in V1.

### Relationship to previous work

Two innovations set the current study apart from those previous. The first was holding fixed several factors between neurophysiological and behavioral measurements: the species and identities of the subjects, the intensity of the display background, the retinal eccentricity of the stimulus, and stimulus size. Two previous primate studies matched these parameters, but neither of them varied temporal frequency, and the one that varied color reported data from few neurons (Jiang et al., 2015a, 2015b; Sperling et al., 1978). A second innovation was the use of a cone current model to quantify information loss through the retina and retinogeniculate synapse (Angueyra and Rieke ,2013; Hass et al., 2015; Horwitz, 2020). Note that macaques are slightly more sensitive than humans to chromatic modulations under a range of conditions (Lindbloom-Brown et al., 2014; Stoughton et al., 2012) and are slightly less sensitive to low-frequency luminance modulations (Gelfand and Horwitz, 2018).

Results from this study are broadly consistent with those of Lee et al. (1990) who compared contrast detection thresholds of human observers to the responses of individual magnocellular-projecting (M) and parvocellular-projecting (P) RGCs. M RGCs responded strongly to luminance modulations and weakly to chromatic modulations. The reverse was true for P RGCs. Individual RGCs of both types were less sensitive than human observers at low frequencies and more sensitive at high frequencies. Results of the current study extend these observations by showing that the sensitivity of LGN populations and observers matches at low temporal frequencies, that the SNR of M and P populations is similar across temporal frequencies at contrast detection threshold, and that retinal circuitry is lossier than cortical circuitry except at high frequencies.

The idea that L − M and L + M temporal contrast sensitivity can be directly related to activity in the M and P pathways has been the subject of much debate. Single-unit recordings are ill-suited for settling this debate because, as shown in this study, many stimuli activate both pathways even at detection threshold. The only stimulus that achieved decisive pathway specificity in this study was the low temporal frequency, L − M stimulus, which modulated parvocellular neurons weakly but exclusively. Low-frequency L + M stimuli modulated magnocellular neurons more strongly than parvocellular neurons, but both populations carried measureable signal. At high frequencies, both magnocellular and parvocellular neurons responded briskly to L + M and L − M stimuli.

### Spatial contrast sensitivity

Visual sensitivity under a range of conditions is bandpass for luminance contrast and low pass for chromatic contrast. Interestingly, this pattern is consistent whether modulations are temporal or spatial. A normative explanation is that L − M signals in natural scenes are small (Ruderman et al., 1998) but important (Carvalho et al., 2017; Rosenthal et al., 2018). Detecting these signals is facilitated by integration (low-pass filtering), a strategy that works over space or time due to the large, stationary nature of objects. L + M signals in natural scenes have greater amplitude, so they can be detected with less integration, permitting finer spatial and temporal resolution and the consequent benefits for visually guided action.

Some mechanisms underlying spatial and temporal visual filtering are shared. For example, low-frequency spatial and temporal modulations are filtered via center-surround RF antagonism (Robson, 1966), and high-frequency modulations are filtered via phototransduction (Cottaris et al., 2020). The spatial effects of phototransduction are linked to small eye movements produced during fixation. A small displacement of a high spatial-frequency grating can stimulate individual cone photoreceptors with contrast increments and decrements close together in time, causing cancellation.

Other mechanisms of spatial and temporal filtering differ, one of which is highlighted by the current results. The current study showed that the temporal filtering of luminance and chromatic modulations is similar in the cortex. In contrast, spatial filtering of luminance and chromatic modulations differs substantially. High spatial frequency luminance sensitivity is limited by midget ganglion cell density, implying near-perfect fidelity of cortical information transmission (Anderson et al., 1991; Banks et al. 1987, 1991; Dacey 1993). Chromatic spatial sensitivity, on the other hand, appears to be limited to a greater degree by cortical processes (Martin et al., 2001; Mullen and Kingdom, 2002; Mullen et al., 2005; Sekiguchi et al., 1993; Solomon et al., 2005) but see (Neitz et al., 2020).

### Caveats

Several disparate data sets were converted to a common SNR metric to facilitate comparison across stages of the visual system. This conversion required mathematical models that could lead to erroneous conclusions if based on erroneous assumptions. The basis of each model, the approximations and assumptions made in their construction, and probable sources of error are discussed below.

### The cone current model

The cone current model was based on patch clamp recordings from *ex vivo* macaque cones under light levels similar to those used in the current study (Angueyra and Rieke, 2013). The model approximates current noise as independent of the signal, which is reasonable at the moderate light levels used in this study (Figure 1 of Angueyra and Rieke, 2013). Cone signaling dynamics were approximated as independent of eccentricity, which is reasonable over the range investigated in this study (2–14°) (Sinha et al., 2017). Absolute detection thresholds predicted by this model are close to those measured behaviorally (Angueyra and Rieke, 2013; Koenig and Hofer, 2011).

Weaknesses of the model include the fact that it is based on a single, canonical temporal impulse response, noise spectrum, and cone distribution, all of which presumably vary across observers (cone distribution does; see Curcio et al., 1987). Indeed, results of this study provide indirect evidence for individual differences. LGN neurons in monkey 2 were more sensitive than those in monkey 1, relative to the cone model (Figure 5). This was true for both magnocellular and parvocellular neurons, consistent with a systematic underestimate of cone sensitivity in monkey 2.

At the highest frequencies tested, magnocellular SNR slightly exceeded the SNR of simulated cone currents in monkey 2 (Figure 5B). This is unrealistic; SNR cannot increase between the cones and the LGN. The population model is not responsible for this discrepancy. The SNR of individual magnocellular neurons from monkey 2 exceeded the SNR of the simulated cones inside their RFs (Figure S4). The number of cones in monkey 2 or the high-frequency sensitivity of the simulated cones may have been underestimated. Cone current simulations were based on recordings made at 4,000–6,500 photoisomerizations per second, whereas cones in the monkeys' eyes during the LGN recording experiments underwent approximately 7,400–8,800 photoisomerizations per second.

### The LGN model

The LGN population model included correlations between neurons of a common type (magnocellular or parvocellular) but not between populations. Consequently, population SNR was computed for magnocellular and parvocellular populations separately. Computing SNR for both populations jointly requires additional assumptions that are ill-constrained by data.

The SNR of each LGN population is a lower bound on the SNR of both of them together. Note that this lower bound approached the theoretical upper bound imposed by the SNR of cone currents at high temporal frequencies (Figure 5). This leads to a prediction: the signals carried by magnocellular and parvocellular neurons with overlapping RFs are largely redundant in response to high temporal frequency modulations. This prediction is consistent with the idea that L − M signals carried by magnocellular neurons derive from the same circuits that mediate cone opponency in midget RGCs (Lee and Sun, 2009; Stockman et al., 2018). It is also consistent with the fact that the responses of midget and parasol RGCs with overlapping RFs share noise that is inherited from the photoreceptors (Ala-Laurila et al., 2011).

### The behavioral model

The behavioral model was based on 13,760 detection trials from monkey 1 and 28,960 from monkey 2. Contrast sensitivity functions predicted from the model (Figure 1A) were similar to those from the literature and to those measured from human subjects performing the same task in the same testing apparatus (Gelfand and Horwitz, 2018; Merigan, 1980; Stavros and Kiorpes, 2008). The model accurately predicted contrast detection thresholds collected after the electrophysiological experiments (Horwitz, 2020). Error in estimated behavioral SNR was approximately 30% (see methods).

### Conclusion

By comparing signal loss upstream and downstream of the LGN under identical conditions, this study showed that the differences between the luminance and chromatic temporal contrast sensitivity functions are due largely to processes upstream of the LGN.

### Limitations of the study

Limitations of this study are discussed in detail in the section *Caveats*. The SNR of LGN spiking responses was estimated from single-unit responses and extrapolated to neuronal populations, a transformation that may be inaccurate. SNR estimates were obtained for magnocellular and parvocellular populations separately, and these estimates were not combined. Cone currents were simulated using a model based on cone light responses recorded *ex vivo*, a preparation which may have affected response properties.

### Resource availability

#### Lead contact

Requests for further information, data, and analysis code should be directed to Greg Horwitz (ghorwitz@uw.edu).

#### Materials availability

This study did not generate new unique reagents.

#### Data and code availability

Data and code generated during this study are available at https://github.com/horwitzlab/LGN-temporal-contrast-sensitivity.

## Methods

All methods can be found in the accompanying transparent methods supplemental file.

## Supplemental information

Document S1. Transparent methods and Figures S1–S4

## Footnotes

Supplemental information can be found online at https://doi.org/10.1016/j.isci.2021.102536.
