# Supplementary material for: Temporal filtering of luminance and chromaticity in macaque visual cortex
Source: iScience. 2021 May 18;24(6):102536. doi: 10.1016/j.isci.2021.102536 (PMC8219838; doi:10.1016/j.isci.2021.102536)
Supplement: Document S1. Transparent methods and Figures S1–S4 [file mmc1.pdf]

iScience, Volume *24*

Supplemental information

Temporal filtering of luminance and chromaticity in macaque visual cortex

Gregory D. Horwitz

SUPPLEMENTAL INFORMATION

Transparent Methods

Experimental Model and Subject Details

Two macaque monkeys (M. mulatta, both male) were used in these experiments. Monkeys 1 and 2 were 13 and 7 years old, respectively, at the time of data collection. All procedures were approved by the University of Washington Institutional Animal Care and Use Committee. Monkeys 1 and 2 in the current study are monkeys 1 and 2 from Gelfand and Horwitz (2018) and from Horwitz (2020). The data analyzed in this report were collected contemporaneously with the data reported in Horwitz (2020) and partially overlap with this previous data set.

Method Details

Behavioral task

Behavioral detection thresholds were measured using a two-alternative, forced-choice contrast detection task (Gelfand & Horwitz 2018). Monkeys sat in a dark room, 61 cm away from a rear-projection screen that was illuminated by a digital light projector (Propixx, VPixx, Inc.) updating at 240 Hz. Stimuli were generated using routines from the Psychophysics Toolbox (Brainard 1997, Kleiner et al. 2007, Pelli 1997). The spectral power distribution of each display primary was characterized with a PR650 spectroradiometer (PhotoResearch, Inc.). The background was an equal-energy white metamer at 130 cd/m^2, producing approximate isomerizations per cone per second of L: 8900, M: 7400, and S: 2300.

The monkey initiated each trial by fixating a 0.2 x 0.2° spot at the center of the screen. An upward-drifting, horizontally oriented Gabor stimulus (1 cycle/° in a 0.15° standard deviation envelope) appeared either in the right or left hemifield. Stimulus contrast increased linearly over 166 ms, remained constant for 334 ms, and then ramped down over 166 ms. After a 100–600 ms delay, the fixation point vanished, and saccade targets appeared 2° to the right and left of fixation. The monkey received a liquid reward for making a saccade to the target in the hemifield in which the Gabor stimulus had appeared. Stimulus contrast, color direction in the LM plane, and temporal frequency varied across randomly interleaved trials, and stimulus location varied across days.

The duration of the stimuli used in this study exceeded psychophysical integration times, which are color- and temporal frequency-dependent (King-Smith & Carden 1976, Rovamo et al. 2003, Rovamo et al. 1996, Smith et al. 1984). A protracted stimulus was necessary to probe low temporal frequencies; low-frequency stimuli cannot be brief.

The data were fit with a model that predicts detection threshold as a function of temporal frequency, color direction in the LM plane, and location in the visual field. This model was used to adjust stimulus contrast to be near the monkeys' detection threshold (Gelfand & Horwitz 2018).

Electrophysiology

Responses of single LGN units were measured with extracellular tungsten microelectrodes (Fredrick Haer, Inc.) and recorded with a Multichannel Acquisition Processor system (Plexon, Inc.). Spike isolation was performed online with SortClient software and refined

offline with OfflineSorter software. Visual fixation was tracked with a scleral search coil (Riverbend Instruments, Inc.) and was required to remain in a 1 x 1° window. Liquid rewards were given for successful fixation. The visual display used in the electrophysiological experiments was identical to that used in the behavioral experiments.

White-noise stimulation

Each recorded LGN neuron was characterized with a white-noise stimulus that consisted of a 10 x 10 grid of 0.2° pixels. The light at each of these pixels was determined by independent, random draws from red, green, and blue Gaussian intensity distributions. The stimulus updated at 60 Hz (every four frames). Spike-triggered averaging was performed online to locate the receptive field of each recorded neuron and offline to classify it as magnocellular or parvocellular.

Near-threshold Gabor stimulation of LGN neurons

Following white noise characterization, each neuron was stimulated with a sequence of Gabor patterns centered on its receptive field. All stimuli were equated for detectability using the model of Gelfand and Horwitz (2018). Each neuron had a unique receptive field location and was therefore probed with a unique set of contrasts. Every stimulus modulated the L- and M-cones of the Stockman, MacLeod, and Johnson 10° standard observer with identical contrasts and did not modulate the S-cones. In randomly interleaved trials, L- and M-cone modulations were in phase, to create an L+M stimulus, or in counterphase, to create an L-M

stimulus.

Quantification and Statistical Analysis

LGN SNR calculation

Firing rate modulations of LGN neurons in response to the stimulus were nearly symmetric around the baseline rate. To quantify these responses, the modulation amplitude of LGN spike trains at the fundamental frequency of the stimulus was extracted from stimulus-present and -absent trials and compared. Both distributions of modulation amplitudes were standardized to make them approximately normal and to reduce their dependence on firing rate (Horwitz 2020). d' was defined as the difference between the means of these two distributions, divided by their pooled standard deviation. Neurometric sensitivity, the contrast at which d' = 1.27, was not measured.

Population d' was defined as the d' for an individual neuron multiplied by a population scale factor (Horwitz 2020). The population scale factor depends on the number of neurons of a given type, magnocellular or parvocellular, that are modulated by the stimulus and is defined as:

$$\text{Population scale factor} = \frac{4\vec{w}^T\vec{\mu}}{\sqrt{4.2\vec{w}^T\Sigma_{pop}^{-1}\vec{w}}}. \qquad \text{eq. 1}$$

where $\vec{\mu}$ is a vector of *n* signals, with one element per neuron in the population, Σ_{pop} is an *n* x *n* covariance matrix representing noise in the population, and $\vec{w} = \Sigma_{pop}^{-1}\vec{\mu}$ is the vector of optimal weights for population read-out. The 4 in the numerator represents the increase in signal obtained by pooling over ON and OFF mosaics in the two eyes. The 4.2 in the denominator

represents the increase in noise incurred through this same pooling and includes a 0.2 that represents additional noise due to anticorrelation between ON and OFF mosaics within each eye (Ala-Laurila et al. 2011, Greschner et al. 2011, Mastronarde 1989).

To calculate $\bar{\mu}$, RFs were modeled as 2-dimensional Gaussian functions truncated at 2 standard deviations. A hexagonal mosaic of RFs was constructed so that each RF touched its six neighbors at the 1 standard deviation boundary (Gauthier et al. 2009). The signal carried by the ith neuron, μ_i, was defined as the integrated product of the stimulus envelope and the ith RF. The RF in the center of the hexagonal array was assumed to correspond to the neuron that was actually recorded. Σ_{pop}, the noise covariance matrix, was constructed by assuming that every neuron was equally noisy and that correlations between neurons were equal to their RF overlap (Ala-Laurila et al. 2011, Trong & Rieke 2008). RF sizes of magnocellular neurons were taken from Derrington & Lennie (1984). Parvocellular RF sizes were taken from Watson (2014) with a 20% reduction in diameter to convert from human to macaque (Dacey & Petersen 1992).

Behavioral SNR calculation

Detection threshold was defined as the contrast needed to support 82% correct choices on the contrast detection task (Gelfand & Horwitz 2018). Decisions in this task can be modeled as comparisons between draws from two independent, homoscedastic, Gaussian distributions. If the draw from the signal distribution exceeds the draw from the noise distribution, the trial is answered correctly. 82% correct is achieved when the means of the two distributions are 1.27 standard deviations apart.

Noisy estimates of detection thresholds, on which this model was based, produce noisy SNR estimates. The average cross-validated prediction error of contrast detection thresholds was 14%. A 14% change in contrast around threshold corresponds to a change from 82% correct to 79–84%, assuming a Weibull psychometric function with a slope of 3 (Wallis et al. 2013). This range corresponds to d' values from 0.89–1.7.

Cone current SNR calculation

The cone current model was developed by Angueyra and Rieke (2013). The implementation used in this study is identical to the one used in (Hass et al. 2015, Horwitz 2020) and is available on GitHub (https://github.com/horwitzlab/LGN-temporal-contrast-sensitivity). Each cone is modeled as a linear temporal filter, the output of which (the signal), is corrupted by additive Gaussian noise. Simulated cone currents were weighted over time and space using a filter identical to the signal. This resulted in two univariate, homoscedastic Gaussian distributions from which d' was calculated (difference in means divided by standard deviation).

Spike train distance-based SNR analysis

The analysis of SNR presented in the main text assumes that LGN signals are at the fundamental frequency of the stimulus (F1). To examine the validity of this assumption, spike train power spectra were computed by discrete Fourier transform (Supplemental Figure 1). Parvocellular neurons, as expected, responded dominantly with an F1-modulated response component, whether the stimulus modulated the L- and M-cones in-phase (Supplemental

Figure 1A) or in counterphase (Supplemental Figure 1B) (Kaplan et al. 1990). The dip in power at approximately 5 Hz is a consistent aspect of parvocellular spike trains even in the absence of contrast in the receptive field (Horwitz 2020).

Magnocellular neurons carry signatures of stimulus frequency in components of their response besides the F1. Frequency-doubled responses (F2) to high-frequency L+M stimuli were pronounced (Supplemental Figure 1C). The magnitude of the F2 component was tightly correlated with the magnitude of the F1 component across neurons within temporal frequency (mean $r = 0.88$, $p < 0.0001$, paired t-test) and across temporal frequencies within neuron (mean $r = 0.81$, $p < 0.0001$, paired t-test). The information carried by the F1 and F2 components is therefore largely redundant. A broadband increase in power at high temporal frequencies is an expected consequence of the rectangular spike counting window and the increase in spike modulation amplitude with temporal frequency (Supplemental Figure 1C & 1D)(Harris 1978).

To ask whether magnocellular spike trains carried stimulus-related signals that were missed by the analysis of F1 modulation, an auxiliary analysis was performed. Each spike train was represented as a point in a high-dimensional space, and the distance between each pair of spike trains was defined on the basis of how many spikes must be added, deleted, or moved to transform one to the other (Victor & Purpura 1997). For each stimulus condition, individual spike trains were extracted and the nine nearest neighbors identified. If five or more of these neighbors were responses to the stimulus, then the extracted spike train was classified as stimulus-present; otherwise, it was classified as stimulus-absent. These classifications were compared to ground truth to quantify correctly classified stimulus responses (hits) and incorrectly classified responses to the blank (false alarms). The signal-to-noise ratio (d') was

calculated as Φ^{-1}(hit rate) - Φ^{-1}(false alarm rate), where Φ^{-1} is the inverse cumulative standard normal probability density function, hit rate is the number of hits divided by the number of stimulus-present trials, and false alarm rate is the number of false alarms divided by the number of stimulus-absent trials.

The results of this analysis agreed closely with the results of the F1-based SNR analysis under most of the conditions tested (Supplemental Figure 2). The one exception was that SNR in response to high-frequency, L-M modulations in monkey 1 was considerably higher under the spike train distance-based analysis than under the F1-based analysis. In this animal, high-frequency L-M modulations often produced transient, weakly entrained responses (e.g. Figure 2B). For the comparisons made in this report, however, the assumption that the signal is carried in the F1 response component is justified.

Spike train distance-based SNR analysis methods

The calculation of SNR based on spike train distances involves two free parameters. The first is the number of nearest neighbors used in the classification, which was set to 9. The second is the penalty associated with moving a spike 1 by second, which was set to 6 (the penalty of adding or subtracting a spike was 1). The values of these two parameters were found via a grid search that maximized decoding accuracy.

Two minor adjustments were made to d' values calculated by this procedure to reduce variability and bias. The first adjustment avoids infinite values that would otherwise occur when a hit rate or a false alarm rate was 0 or 1. Zeros were replaced with *0.5/n* and ones were replaced with *(n-0.5)/n*, where *n* is the number trials, either stimulus-present or -absent

(Stanislaw & Todorov 1999). The second correction compensates for a small downward bias produced by the fact that each trial was classified on the basis of other trials, which are likely to be of the opposite type. Consider an urn containing equal numbers of red and black balls: the nearest neighbors of a red ball, not including itself, are more likely to be black than red. To correct for this fact, Φ^{-1} (E(hit rate)) - Φ^{-1} (E(false alarm rate)) was subtracted from d', where E(hit rate) and E(false alarm rate) are the expected values of the hit rate and false alarm rate under the ball and urn model.

KEY RESOURCE TABLE

REAGENT or RESOURCE	SOURCE	IDENTIFIER
Rhesus monkeys (Macaca mulatta)	Washington National Primate Research Center	N/A
MATLAB	Mathworks	https://www.mathworks.com/products/matlab.html; RRID: SCR_001622
Plexon Sort Client	Plexon, Inc.	http://www.plexon.com; RRID: SCR:003170
Plexon Offline Sorter	Plexon, Inc.	http://www.plexon.com; RRID: SCR:003170

Supplemental Figure legends

Figure S1. Spectral analysis of LGN spike trains, related to Figure 2. Power spectra are shown for parvocellular responses to L+M stimuli (**A**), parvocellular responses to L-M stimuli (**B**), magnocellular responses to L+M stimuli (**C**), and magnocellular responses to L-M stimuli (**D**). The fundamental frequency of each stimulus is shown along the abscissa (triangles). Frequency-doubled responses in (C) are indicated by black arrows.

Figure S2. Magnocellular signal-to-noise ratio analysis, related to Figure 2. Signal-to-noise ratio (d') was computed in response to L+M modulations (black) and L-M modulations (magenta) at the fundamental frequency of the stimulus (closed symbols) and from the performance of a k-nearest neighbors spike train classifier (open symbols). Symbols represent means across neurons, and shaded bands represent ± 1 standard error of the mean. Data are from monkey 1 (**A**) and monkey 2 (**B**).

Figure S3. Analysis of spike counting window on population signal-to-noise ratio, related to Figure 4. Population signal-to-noise ratio was calculated from parvocellular responses to L-M modulations (magenta) and magnocellular responses to L+M modulations (black). Spikes were counted for 0.05, 0.1, 0.2, 0.4, or 0.66 s (columns), starting 0, 0.05, 0.1, 0.15, 0.2, 0.25, or 0.3 s after stimulus onset (rows). Symbols represent means across neurons, and shaded bands

represent ±1 standard error of the mean. Horizontal lines indicate signal-to-noise ratios of 0 (gray) and 1.27 (dashed).

Figure S4. Signal-to-noise ratio of individual LGN neurons and the cones assumed to be inside their receptive fields, related to Figure 5. Plotting conventions are as in Figure 5, but population scaling has not been performed on the individual neuronal d' values, and only the cones inside the receptive field of each recorded LGN neuron were simulated.

SUPPLEMENTAL REFERENCES

Ala-Laurila P, Greschner M, Chichilnisky EJ, Rieke F. 2011. Cone photoreceptor contributions to noise and correlations in the retinal output. *Nat Neurosci* 14: 1309-16

Angueyra JM, Rieke F. 2013. Origin and effect of phototransduction noise in primate cone photoreceptors. *Nat Neurosci* 16: 1692-700

Brainard DH. 1997. The Psychophysics Toolbox. *Spat Vis* 10: 433-6

Dacey DM, Petersen MR. 1992. Dendritic field size and morphology of midget and parasol ganglion cells of the human retina. *Proc Natl Acad Sci U S A* 89: 9666-70

Derrington AM, Lennie P. 1984. Spatial and temporal contrast sensitivities of neurones in lateral geniculate nucleus of macaque. *J Physiol* 357: 219-40

Gauthier JL, Field GD, Sher A, Shlens J, Greschner M, et al. 2009. Uniform signal redundancy of parasol and midget ganglion cells in primate retina. *The Journal of neuroscience : the official journal of the Society for Neuroscience* 29: 4675-80

Gelfand EC, Horwitz GD. 2018. Model of parafoveal chromatic and luminance temporal contrast sensitivity of humans and monkeys. *J Vis* 18: 1

Greschner M, Shlens J, Bakolitsa C, Field GD, Gauthier JL, et al. 2011. Correlated firing among major ganglion cell types in primate retina. *J Physiol* 589: 75-86

Harris FJ. 1978. On the use of windows for harmonic analysis with the discrete Fourier transform *Proceedings of the IEEE* 66: 51-83

Hass CA, Angueyra JM, Lindbloom-Brown Z, Rieke F, Horwitz GD. 2015. Chromatic detection from cone photoreceptors to V1 neurons to behavior in rhesus monkeys. *J Vis* 15: 1

Horwitz GD. 2020. Temporal information loss in the macaque early visual system. *Plos Biology* 18

Kaplan E, Lee BB, Shapley R. 1990. New views of primate retinal function. *Progress in retinal research* 9: 273-336

King-Smith PE, Carden D. 1976. Luminance and opponent-color contributions to visual detection and adaptation and to temporal and spatial integration. *J Opt Soc Am* 66: 709-17

Kleiner M, Brainard DH, Pelli DG. 2007. *What's new in Psychtoolbox-3*. Presented at Perception 36 ECVP Abstract Supplement

Mastronarde DN. 1989. Correlated firing of retinal ganglion cells. *Trends Neurosci* 12: 75-80

Pelli DG. 1997. The VideoToolbox software for visual psychophysics: transforming numbers into movies. *Spat Vis* 10: 437-42

Rovamo J, Kukkonen H, Raninen A, Donner K. 2003. Efficiency of temporal integration of sinusoidal flicker. *Invest Ophthalmol Vis Sci* 44: 5049-55

Rovamo J, Raninen A, Lukkarinen S, Donner K. 1996. Flicker sensitivity as a function of spectral density of external white temporal noise. *Vision Res* 36: 3767-74

Smith VC, Bowen RW, Pokorny J. 1984. Threshold temporal integration of chromatic stimuli. *Vision Res* 24: 653-60

Stanislaw H, Todorov N. 1999. Calculation of signal detection theory measures. *Behav Res Methods Instrum Comput* 31: 137-49

Trong PK, Rieke F. 2008. Origin of correlated activity between parasol retinal ganglion cells. *Nat Neurosci* 11: 1343-51

Victor JD, Purpura KP. 1997. Metric-space analysis of spike trains: Theory, algorithms and application. *Network-Computation in Neural Systems* 8: 127-64
Wallis SA, Baker DH, Meese TS, Georgeson MA. 2013. The slope of the psychometric function and non-stationarity of thresholds in spatiotemporal contrast vision. *Vision Res* 76: 1-10
Watson AB. 2014. A formula for human retinal ganglion cell receptive field density as a function of visual field location. *J Vis* 14

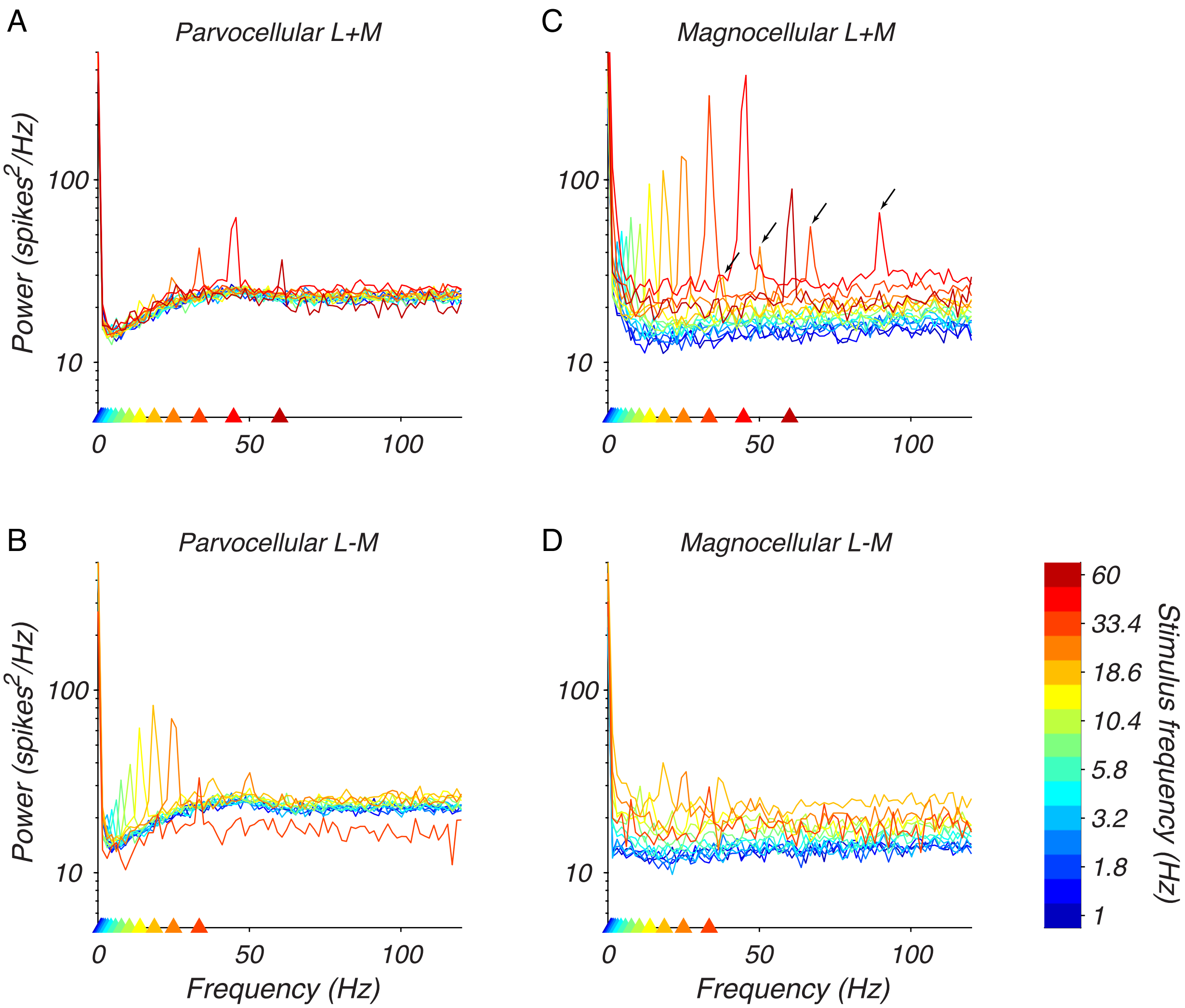

A
Parvocellular L+M
C
Magnocellular L+M
B
Parvocellular L-M
D
Magnocellular L-M
Power (spikes²/Hz)
Frequency (Hz)
Stimulus frequency (Hz)
60
33.4
18.6
10.4
5.8
3.2
1.8
1

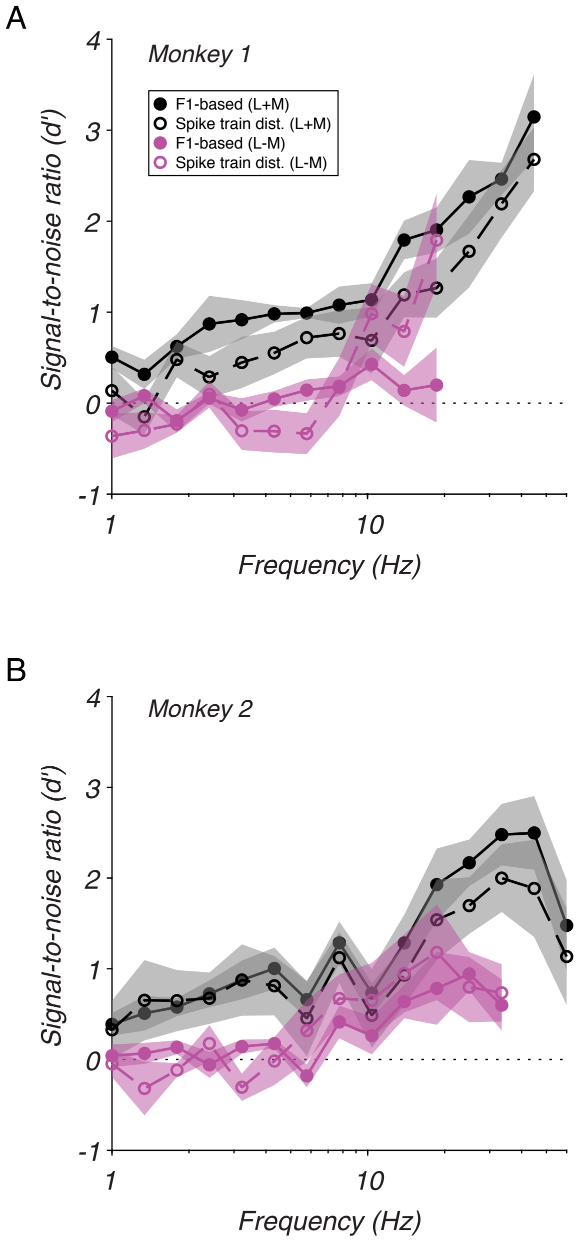
A
Monkey 1
F1-based (L+M)
Spike train dist. (L+M)
F1-based (L-M)
Spike train dist. (L-M)
Signal-to-noise ratio (d')
4
3
2
1
0
-1
1
10
Frequency (Hz)
B
Monkey 2
Signal-to-noise ratio (d')
4
3
2
1
0
-1
1
10
Frequency (Hz)

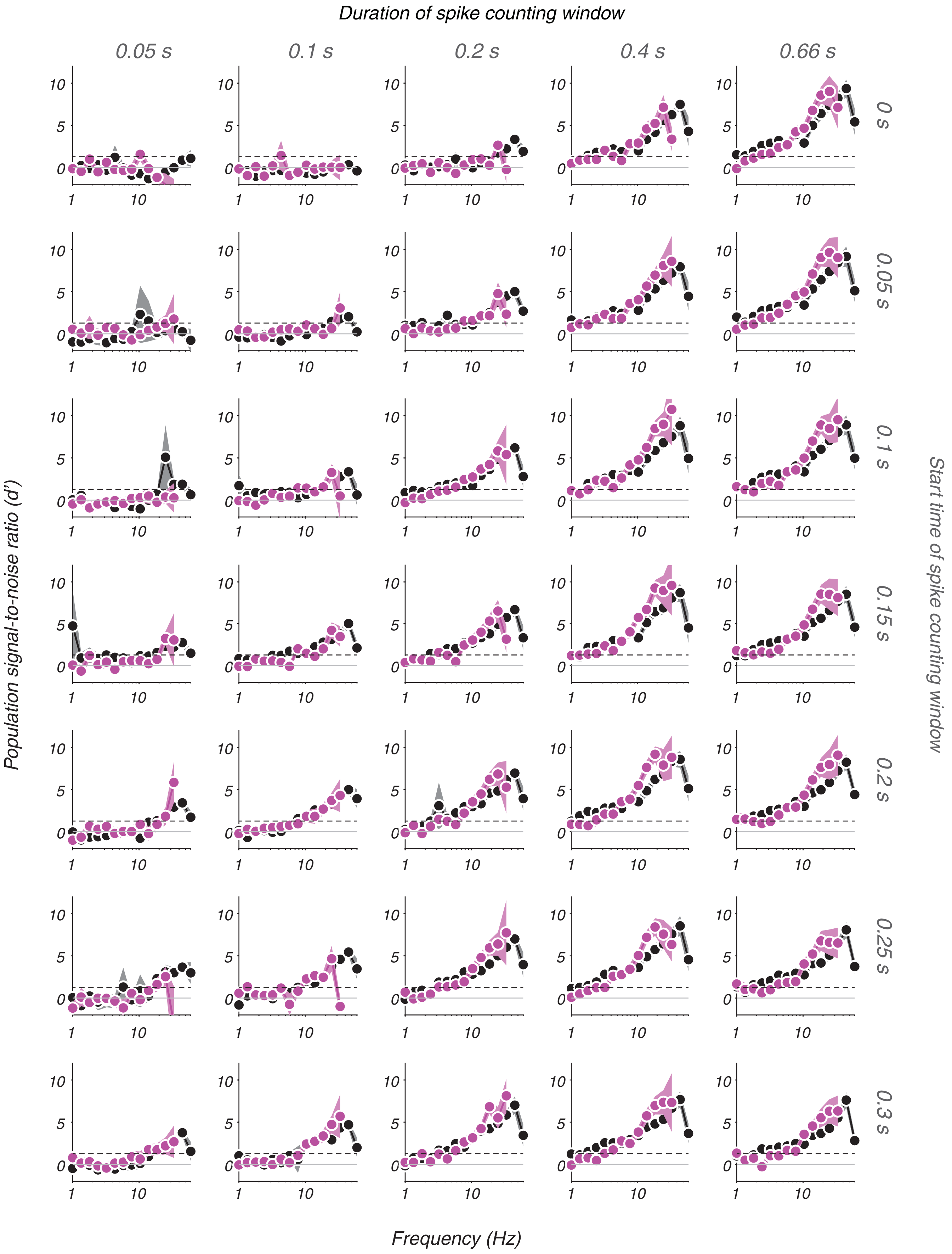
Duration of spike counting window
0.05 s
0.1 s
0.2 s
0.4 s
0.66 s
0 s
0.05 s
0.1 s
0.15 s
0.2 s
0.25 s
0.3 s
Start time of spike counting window
Population signal-to-noise ratio (d')
Frequency (Hz)

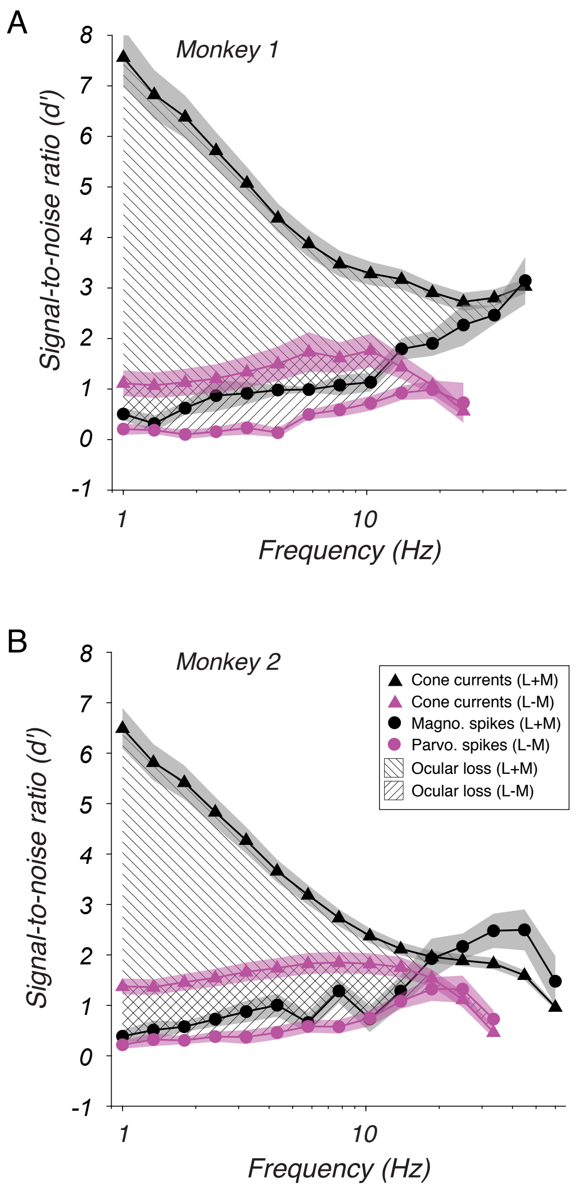
A
Monkey 1
Signal-to-noise ratio (d')
Frequency (Hz)
B
Monkey 2
Cone currents (L+M)
Cone currents (L-M)
Magno. spikes (L+M)
Parvo. spikes (L-M)
Ocular loss (L+M)
Ocular loss (L-M)
Signal-to-noise ratio (d')
Frequency (Hz)
